# Evaluation of 41 single nucleotide polymorphisms in canine diffuse large B-cell lymphomas using MassARRAY

**DOI:** 10.1038/s41598-022-09112-0

**Published:** 2022-03-24

**Authors:** Sirintra Sirivisoot, Tanit Kasantikul, Somporn Techangamsuwan, Araya Radtanakatikanon, Ken Chen, Tzu-yin Lin, Anudep Rungsipipat

**Affiliations:** 1 Center of Excellence for Companion Animal Cancer, Department of Pathology, Faculty of Veterinary Science, Chulalongkorn University, Bangkok, Thailand; 2 Clemson Veterinary Diagnostic Center, Clemson University, Columbia, SC USA; 3 SQ Reference Lab, Beiqing Road, Qingpu district, Shanghai, China; 4 University of California Davis, Sacramento, CA USA

**Keywords:** Cancer genetics, Lymphoma

## Abstract

Diffuse large B-cell lymphoma (DLBCL) is the most common subtype of lymphoma in dogs with a multicentric form. This study aimed to assemble 41 variants of the previously reported genes and to investigate these variants in canine DLBCL using the Agena MassARRAY platform. These variants were chosen based on the high prevalence observed in canine B- and T-cell lymphomas, their significance for target therapy, and compatibility for multiplex PCR amplification. Lymph node biopsy was performed from 60 dogs with B-cell lymphoma comprising 47 purebred and 13 crossbred dogs. All dogs presented single nucleotide polymorphisms (SNPs) at *HYAL4* and *SATB1* genes. The lesser mutual SNPs were observed at *SEL1L*, excluding a cocker spaniel, and *c-Kit*, with the exception of a pug and a French bulldog. Even though no statistical association was noted between each SNP and dog breed, purebreds were 3.88 times more likely to have a SNP at FLT3 rs852342480 (95%CI 0.50–45.03, *p* = 0.26), 3.64 times at TRAF3 F306X (95%CI 0.58–42.50, *p* = 0.43) and 2.66 times at TRAF3 E303EX (95%CI 0.56–13.12, *p* = 0.31). Also, DLBCL dogs (CHOP-based treatment) with *c-Kit* T425= had a poorer prognosis with shorter median overall survival times (OST) than dogs with the wild type. Dogs treated with COP chemotherapy and contained 3–5 variants at *SEL1L* were associated with decreased median OST. Therefore, this SNP’s lymphoma panel provides valuable information that we can use to outline a prognosis and develop a treatment plan for the targeted therapy of each dog.

## Introduction

Multicentric lymphoma is the most relevant anatomical form in dogs. Based on cellular morphology, cell lineage and topography, the most common subtypes of B-cell lymphoma in dogs are diffuse large B-cell lymphoma (DLBCL) and marginal zone lymphoma (MZL)^1^. Peripheral T-cell lymphoma (PTCL) and T-zone lymphoma (TZL) are frequently reported as T subtypes^1^. Both MZL and TZL are low-grade and have indolent behaviors that cause a low mortality rate^2^. In contrast, high-grade lymphomas such as DLBCL and PTCL are related to poorer prognoses and a shorter survival time^3^. The CHOP (cyclophosphamide, doxorubicin, vincristine, and prednisolone) protocol is the standard-of-care in dogs with naïve intermediate- and high-grade lymphoma^4^; however, refractory and relapsed diseases present major barriers to successful treatment. Therefore, novel strategies such as immunotherapy and target drugs have been investigated to enhance the effectiveness of therapy in canine lymphomas^5^. An autologous heat shock protein peptide chaperone (HSPPC) vaccine was evaluated the efficacy of antitumor effect in dogs with B-cell lymphoma treated with CHOP-based protocol. Vaccinated dogs compared to placebo group showed promising results which prolonged time to progression and lymphoma-specific survival with no local or systemic adverse effects^6,7^. Anti-canine CD20 monoclonal antibody has also been developed for treating B-cell lymphomas. The generation of this chimeric antibody against CD20 showed good binding affinity to canine B cells and induced antibody-dependent cell-mediated cytotoxicity (ADCC) against canine neoplastic B cells in vitro and in vivo studies^8–10^. Small molecules such as NEMO-binding domain (NBD) peptide and NEDD-8 activating enzyme inhibitor (Pevonedistat) inhibited nuclear factor kappa-B (NF-kB) activity, commonly upregulated in B-cell lymphoma, and induced apoptosis of malignant B lymphocytes^11,12^. This might be useful for future clinical application in lymphoma dogs that have genetic mutation in the NF-kB pathway.

Gene expression analysis and next-generation sequencing technology have been used to investigate the potential risk genes and probable signaling pathways that may cause specific diseases and cancers. In humans, global gene expression profiling has indicated two subtypes of DLBCL: neoplastic cells that derive from germinal center B cells (GCB DLBCL) or those from post-germinal activated B cells (ABC DLBCL)^13^. The ABC subgroup is notably stimulated through B-cell receptors including many of the NF-kB target genes^14^, whereas genetic mutations in GCB DLBCL are frequently observed in chromatin modifiers and histone proteins such as *KMT2D*, *MYD88*, *CARD11*, *EZH2* and *CREBBP*^15,16^. Interestingly, Richards et al.^17^ investigated gene expression profiling in canine B-cell lymphomas and found similarities to humans. Based on the immunohistochemistry and gene expression pattern, two distinct groups were classified as the ABC-like and the GCB-like DLBCL. The GCB-like group had higher expression of *IRAK1BP1* and *STAT4*, while the ABC type had increased expression of NF-kB pathway genes. Furthermore, the canine ABC-like group had significantly poorer the progression-free and overall survival times compared to the GCB subgroup resembling with human DLBCL.

Several studies currently have used whole genome, whole exome and whole transcriptome (RNA-Seq) sequencing to explore the aberrant genes contributing to lymphomagenesis in specific dog breeds. Elvers et al.^18^ investigated the genetic risk factors in three lymphoma-predisposed breeds: boxers for T cells, cocker spaniels for B cells and golden retrievers for B and T cells. The authors found strong similarities between the mutations in both dog breeds with B-cell lymphoma, occurring in *TRAF3-MAP3K14*, *FBXW7* and *POT1*. However, the boxer with T-cell lymphoma typically had mutations in the *PTEN-mTOR* pathway, which was dissimilar to the golden retriever with T-cell lymphoma that usually exhibited mutations in genes related to cellular metabolism. In addition to these findings, multiple somatic point mutations were identified in canine B-cell lymphoma including *TRAF3*, *POT1*, *LMNB1* and *MVB12A*^19–21^. Recently, single nucleotide polymorphisms (SNPs) that affected *SPAM1*, *HYAL4*, *HYLAP1*, *PTEN* and *SATB1* were noted in canine T-cell lymphomas^22–24^.

Even though fresh frozen tissue is more preferable for genetic mutation analysis due to conserving DNA quality, formalin-fixed paraffin embedded (FFPE) technique is a standard method in routine work in the pathology unit. The FFPE tissue could preserve the cellular morphology and keep at room temperature for several years. However, the fixation process usually causes DNA cross-linkage, degradation, and fragmentation which could interfere the accuracy of molecular studies especially RNA^25^. There are several studies have compared the DNA quality between fresh and FFPE specimens by using next generation sequencing. The results of mutation analysis from those two types of samples were comparable^26^. Moreover, FFPE tissues from dogs with call rate > 65% provided similar results of single nucleotide variation when compared to fresh whole blood samples^27^.

In the present study, we assembled a custom SNP panel of the previously reported genes that may drive lymphomagenesis in dogs. The criteria for variants selection were high prevalence in specific dog breeds, application for target therapy, and primer compatibility for multiplex polymerase chain reaction (PCR) with maximum ability of MassARRAY. This lymphoma SNP panel was then investigated in dogs with DLBCL, studying 47 purebred and 13 crossbred dogs, from archival FFPE samples. Beyond SNPs’ evaluation, the mutation genotyping panel was found to be different in each dog. This might be of relevance when it comes to outlining prognoses and selecting targeted therapies for affected dogs, by contributing toward increasing the treatment efficacy of personalized medicine.

## Methods

### Tissue samples and immunohistochemistry

Formalin-fixed paraffin-embedded (FFPE) tissues were obtained from the archive of the Department of Pathology, Faculty of Veterinary Science, Chulalongkorn University and SQ Reference Lab, China between 2011 and 2021. A statement to confirm that all methods were carried out in accordance with relevant guidelines, regulations and sampling procedures was approved by the Chulalongkorn University Animal Care and Use Committee**.**

All dogs had a history of generalized lymphadenopathy and were diagnosed as having nodal lymphomas based on the cytopathology and histopathology. Lymph node samples were collected at the time of presentation and before receiving chemotherapy. Immunostaining with CD3, CD20 and Pax5 and/or a clonality test were performed for lymphoma subtype classification following REAL/WHO (Revised European American Lymphoma/World Health Organization)^1^. The immunohistochemistry for CD20, CD3 and Pax5 was adapted from a previous study^28^. Briefly, tissue section was quenched endogenous peroxidase by 0.3% (v/v) H_2_O_2_ for 30 min and 5% bovine serum albumin was used for non-specific blocking for 20 min. Antigen was unmasked for CD3 and Pax5 by 10 mM citrate buffer (pH 6) in water bath at 95 °C for 20 min. Then, the slide was incubated with primary antibodies: CD20 (1:300, ab27093, Abcam, MA, USA), CD3 (1:10, PF. Moore, CA, USA), and Pax5 (1:100, 1EW, Leica, Newcastle Upon Tyne, UK) for 1.5 h. Secondary antibodies were applied for 30 min using EnVision system-HRP, mouse/rabbit (Dako, Glostrup, Denmark) for CD20 and Pax5 and ImmPRESS HRP, rat (Vector Laboratories Inc., CA, USA) for CD3. Visualization system for CD20 and Pax5 was DAB (Dako) and for CD3 was NovaRED (Vector Laboratories Inc.) as a substrate. Only DLBCL was included in this study. Demographic information was also recorded for each subject (Table 1). From 60 dogs, 26 dogs were treated with COP-based protocol (cyclophosphamide, vincristine, and prednisolone), 12 dogs received CHOP-based protocol, and the rest were loss of contact or death after diagnosis.

**Table 1:** Demographics of 60 diffuse large B-cell lymphoma dogs in this study.

	ST (11)	GR (12)	LR (5)	Beagle (2)	BT (2)	Corgi (2)	FB (2)	Poodle (2)	Pug (2)	WHWT (2)	CHH (1)	CP (1)	GP (1)	JRT (1)	Pom (1)	Mixed (13)
Male	5	7	1	1	1	1	1	1	–	1	1	–	1	–	1	4
Mc	2	–	2	–	–	1	–	–	1	–	–	–	–	–	–	3
Female	4	2	1	–	1	–	1	–	–	1	–	–	–	1	–	5
Fs	–	3	1	1	–	–	–	1	1	–	–	1	–	–	–	1
Mean age (range)	11.1 (7–14)	10.4 (7–14)	8.5 (3–11)	11.5 (10–13)	7	5	7 (5–9)	9 (7–11)	5 (4–6)	12 (11–13)	7	15	7	9	5	9.1 (2–13)

*BT* Bull terrier, *CHH* Chihuahua, *CP* Cocker spaniel, *FB* French bulldog, *Fs* sprayed female, *GP* German shepherd, *GR* Golden retriever, *JRT* Jack Russel terrier, *LR* Labrador retriever, *Mc* castrated male, *Pom* Pomeranian, *ST* Shih Tzu, *WHWT* West highland white terrier.

### DNA extraction

Each FFPE block was shaved to 75–100 µm thickness in a sterile microcentrifuge tube. The samples were then deparaffinized with xylene and absolute ethanol, respectively, before genomic DNA extraction using a DNeasy Blood and Tissue Kit (Qiagen, Hilden, Germany) following manufacturer’s instructions. The DNA concentration from each sample was quantified using a Nanodrop Lite Spectrophotometer (Thermo Scientific, MA, USA) and kept at -20 °C until use. The DNA samples were required to have a level of quality marked by at least a 260/280 absorbance ratio between 1.5 and 2.0 and concentration ≥ 5 ng/µl, when examined using a MassARRAY analyzer (Agena Bioscience, CA, USA).

### Gene polymorphisms’ detection

Forty-one SNPs, in the mutated genes of dogs with lymphoma, were selected from previous studies (Table 2) based on the incidence in various dog breeds such as golden retriever, cocker spaniel, and mixed breed. The nucleotide locations were defined according to the sequence provided in the CanFam 3.1 reference genome (www.ensembl.org). The primer designed process was followed the manufacturer's online specific software for MassARRAY system, AgenaCx (Agena Bioscience). The compatibility of multiple primer sets were selected to avoid primer dimer and their concentrations with efficient amplification were used for the multiplex PCR. Each primer pair had repeatability from 85 to 100%. The multiplex PCR cocktail comprised 0.5 μL of PCR buffer (10X), 0.4 μL MgCl_2_ (25 mM), 0.1 μL dNTPs (25 mM), 0.2 μL PCR enzyme (5 U/μL), 1 μL amplification primer mix, 2 μL DNA (20 ng/μL) and HPLC-grade H_2_O to a total volume of 5 μL. The thermocycling conditions were 95 °C for 2 min followed by 45 cycles at 95 °C for 30 s, 56 °C for 30 s and 72 °C for 1 min, with a final incubation at 72 °C for 5 min.

**Table 2:** The prevalence of 41 variant lists in 60 canine diffuse large B-cell lymphomas and dog breeds.

Genes (Reference)	Variants	Position	Amino acid change	Allele	Number of positive mutation /Totals read dog (%)	ST N = 11	GR N = 12	LR N = 5	Mixed N = 13	Other pure breeds N = 19
*C-kit*^36^	rs22299980 A > G	Chr13:47,175,092	p.Thr425 =	G	**28/58** (48.28%)	6/10	5/12	2/5	6/13	9/18
			p.Asn426GlufsTer25	AG	**16/58** (27.58%)	3/10	3/12	2/5	6/13	2/18
			Wild type	A	14/58 (24.14%)	1/10	4/12	1/5	1/13	7/18
*FLT3*^36^	c.1647 + 10A > C	Chr25:11,645,948	Intron variant	C	1/60 (1.67%)	1/11	0/12	0/5	0/13	0/19
				AC	1/60 (1.67%)	1/11	0/12	0/5	0/13	0/19
			Wild type	A	58/60 (96.67%)	9/11	12/12	5/5	13/13	19/19
	rs852342480 A > G	Chr25:11,646,177	Intron variant	G	7/58 (12.1%)	0/11	0/12	1/5	1/12	5/18
				GA	6/58 (10.34%)	0/11	2/12	1/5	0/12	3/18
			Wild type	A	45/58 (77.58%)	11/11	10/12	3/5	11/12	10/18
	rs23257447 T > C	Chr25:11,658,204	Intron variant	C	4/38 (10.53%)	0/5	0/11	0/2	0/8	4/12
				CT	2/38 (5.26%)	0/5	0/11	0/2	1/8	1/12
			Wild type	T	32/38 (84.21%)	5/5	11/11	2/2	7/8	7/12
*POT1*^21^	c.850C > T	Chr14:11,033,690	p.Arg284Cys	T	4/59 (6.78%)	0/11	0/12	1/5	1/12	2/19
			p.Arg284LeufsTer19	CT	2/59 (3.38%)	1/11	0/12	0/5	0/12	1/19
			Wild type	C	53/59 (89.83%)	10/11	12/12	4/5	11/12	16/19
	c.927del	Chr14:11,033,763	p.Phe309LeufsTer3	Del	8/60 (13.33%)	2/11	1/12	0/5	2/13	3/19
			Wild type	T	52/60 (86.67%)	9/11	11/12	5/5	11/13	16/19
	c.1747C > T	Chr14:11,053,090	p.Arg583Ter	T	3/60 (5%)	0/11	0/12	0/5	0/13	3/19
			Wild type	C	57/60 (95%)	11/11	12/12	5/5	13/13	16/19
	c.1928 T > C	Chr14:11,056,601	p.Phe643Ser	C	2/59 (3.38%)	0/10	0/12	1/5	0/13	1/19
			Wild type	T	57/59 (96.61%)	10/10	12/12	4/5	13/13	18/19
*TRAF3*^21^	rs851689319 A > T	Chr8:70,782,945	p.Lys284Ter	T	15/57 (26.32%)	3/10	2/11	2/5	2/12	6/19
			p.Lys284IlefsTer14	AT	2/57 (3.51%)	0/10	0/11	0/5	1/12	1/19
			Wild type	T	40/57 (70.18%)	7/10	9/11	3/5	9/12	12/19
	c.906delT	Chr8:70,782,999	p.Ile302MetfsTer21	Del	13/56 (23.21%)	3/10	2/12	2/5	1/11	5/18
			Wild type	T	43/56 (76.79%)	7/10	10/12	3/5	10/11	13/18
	c.908dupA	Chr8:70,783,003	p.Arg304GlufsTer9	A	16/56 (28.57%)	5/11	4/11	1/4	2/13	4/17
			Wild type	Del	40/56 (71.43%)	6/11	7/11	3/4	11/13	13/17
	c.968_971delACAG	Chr8:70,788,018	p.Ile323ThrfsTer7	Del	4/58 (6.9%)	2/10	0/11	0/5	1/13	1/19
			Wild type	ACAG	54/58 (93.1%)	8/10	11/11	5/5	12/13	18/19
	c.942_949dupCCAAAATA	Chr8:70,783,043	p.Leu317ProfsTer9	CCAAAATA	3/49 (6.12%)	1/7	1/12	0/4	0/10	1/16
			Wild type	Del	46/49 (93.88%)	6/7	11/12	4/4	10/10	15/16
	c.1591_1592insTC	Chr8:70,789,530	p.Ala531ValfsTer14	TC	0/59 (0%)	0/11	0/12	0/5	0/13	0/18
			Wild type	Del	59/59 (100%)	11/11	12/12	5/5	13/13	18/18
	c.1195delC	Chr8:70,789,131	p.Leu399TrpfsTer20	Del	0/60 (0%)	0/11	0/12	0/5	0/13	0/19
			Wild type	C	60/60 (100%)	11/11	12/12	5/5	13/13	19/19
	c.1652delA	Chr8:70,789,589	p.Asp551ValfsTer9	Del	0/54 (0%)	0/10	0/12	0/5	0/11	0/16
			Wild type	A	54/54 (100%)	10/10	12/12	5/5	11/11	16/16
	c.1339delA	Chr8:70,789,276	p.Thr447ArgfsTer14	Del	0/59 (0%)	0/10	0/12	0/5	0/13	0/19
			Wild type	A	59/59 (100%)	10/10	12/12	5/5	13/13	19/19
	c.966_979del AGTAATAGACAGCC	Chr8:70,788,012- 70,788,026	p.Ile323ArgfsTer69	Del	0/58 (0%)	0/10	0/12	0/4	0/13	0/19
			Wild type	AGTAATAGACAGCC	58/58 (100%)	10/10	12/12	4/4	13/13	19/19
	c.1434_1445del ATGCGTGGAGAG	Chr8:70,789,371–70,789,383	p.Met478_Tyr482delinsIle	Del	0/60 (0%)	0/11	0/12	0/5	0/13	0/19
			Wild type	ATGCGTGGAGAG	60/60 (100%)	11/11	12/12	5/5	13/13	19/19
*HYAL4*^23^	rs8499846 G > A	Chr14:11,778,977	Upstream gene variant	A	**25/54** (46.3%)	6/10	1/10	2/5	6/13	10/16
				GA	**13/54** (24.1%)	3/10	5/10	1/5	4/13	0/16
			Wild type	G	16/54 (29.63%)	1/10	4/10	2/5	3/13	6/16
	14:11791385 A > G	Chr14:11,791,385	Intergenic variant	G	**11/60** (18.3%)	1/11	0/12	0/5	1/13	9/19
				GA	**28/60** (46.67%)	5/11	8/12	2/5	7/13	6/19
			Wild type	A	21/60 (35%)	5/11	4/12	3/5	5/13	4/19
	14:11794735 C > T	Chr14:11,794,735	Intergenic variant	T	**18/60** (30%)	1/11	1/12	0/5	6/13	10/19
				CT	**23/60** (38.33%)	5/11	7/12	2/5	4/13	5/19
			Wild type	C	19/60 (31.67%)	5/11	4/12	3/5	3/13	4/19
	14:11807161 G > A	Chr14:11,807,161	Intergenic variant	A	**7/60** (11.67%)	0/11	2/12	0/5	1/13	4/19
				GA	**20/60** (33.33%)	2/11	4/12	1/5	5/13	8/19
			Wild type	G	33/60 (55%)	9/11	6/12	4/5	7/13	7/19
*SATB1*^18,22^	c.1259A > C	Chr23:24,651,976	p.Gln420Pro	C	**58/60** (96.67%)	9/11	12/12	5/5	13/13	19/19
			p.Glu420ProfsTer20	CA	**1/60** (1.67%)	1/11	0/12	0/5	0/13	0/19
			Wild type	A	1/60 (1.67%)	1/11	0/12	0/5	0/13	0/19
*SEL1L*^23^	8:53778185 T > C	Chr8:53,778,185	Intron variant	C	**25/59** (42.37%)	2/11	9/12	1/4	4/13	9/19
				CT	**19/59** (32.2%)	5/11	2/12	2/4	5/13	5/19
			Wild type	T	15/59 (25.42%)	4/11	1/12	1/4	4/13	5/19
	8:53785948 A > G	Chr8: 53,785,948	Intron variant	G	**8/51** (15.68%)	1/10	4/10	0/4	1/12	2/15
				GA	**12/51** (23.53%)	2/10	2/10	0/4	4/12	4/15
			Wild type	A	31/51 (60.78%)	7/10	4/10	4/4	7/12	9/15
	rs24507594 G > A	Chr8:53,796,442	Intron variant	A	**18/54** (33.3%)	2/10	8/11	1/4	3/13	4/16
				GA	**16/54** (29.63%)	5/10	2/11	2/4	4/13	3/16
			Wild type	G	20/54 (37%)	3/10	1/11	1/4	6/13	9/16
	8:53818371 G > A	Chr8:53,818,371	Intron variant	A	**11/26** (42.31%)	2/5	3/4	2/2	0/7	4/8
				AG	**4/26** (15.38%)	2/5	1/4	0/2	1/7	0/8
			Wild type	G	11/26 (42.31%)	1/5	0/4	0/2	6/7	4/8
	rs24560262 C > A	Chr8:52,763,337	Intergenic variant	A	**19/60** (31.67%)	6/11	6/12	1/5	3/13	3/19
				CA	**18/60** (30%)	2/11	3/12	3/5	5/13	5/19
			Wild type	C	23/60 (38.33%)	3/11	3/12	1/5	5/13	11/19
*SPAM1*^23^	c.1445 T > A	Chr14:11,704,952	p.Lys482Met	A	2/58 (3.45%)	0/11	0/10	1/5	0/13	1/19
	c.1445 T > G		p.Lys482Thr	G	0/58 (0%)	0/11	0/10	0/5	0/13	0/19
	rs851582160 T > C		p.Lys482Arg	C	13/58 (22.41%)	2/11	6/10	1/5	2/13	2/19
			p.Lys482ArgfsTer27	TC	10/58 (17.24%)	1/11	2/10	1/5	5/13	1/19
			Wild type	T	33/58 (56.9%)	8/11	2/10	2/5	6/13	15/19
*TP53*^24^	rs852661628 G > A	Chr5:32,563,389	p.Arg301Trp	A	1/58 (1.72%)	0/10	0/11	1/5	0/13	0/19
			Wild type	G	57/58 (98.28%)	10/10	11/11	4/5	13/13	19/19
*MVB12A*^20^	c.361C > T	Chr20:45,367,953	p.Asp121Asn	T	0/60 (0%)	0/11	0/12	0/5	0/13	0/19
			p.Asp121ArgfsTer81	CT	2/60 (3.33%)	0/11	1/12	0/5	1/13	0/19
			Wild type	C	58/60 (96.67%)	11/11	11/12	5/5	12/13	19/19
*ZC3H7A*^20^	c.2792A > G	Chr6:31,068,652	p.His931Arg	G	9/10 (90%)	2/2	1/1	1/2	2/2	3/3
			Wild type	A	1/10 (10%)	0/2	0/1	1/2	0/2	0/3
*ZNHIT6*^20^	c.-14G > C	Chr6:62,335,585	5’ UTR variant	GC	6/60 (10%)	2/11	0/12	0/5	2/13	2/19
			Wild type	G	54/60 (90%)	9/11	12/12	5/5	11/13	17/19
*MET*^24^	c.3804C > G	Chr14: 55,699,186	p.Asp1268Glu	G	3/40 (7.5%)	0/6	1/10	1/2	0/7	1/15
			p.Asp1268GlufsTer4	GC	1/40 (2.5%)	0/6	0/10	1/2	0/7	0/15
			Wild type	C	36/40 (90%)	6/6	9/10	0/2	7/7	14/15
*PTEN*^18,22^	rs397513087 C > T	Chr26:37,910,068	p.Leu325 =	T	2/41 (4.88%)	1/7	0/8	0/4	1/9	0/13
			p.Asp326ArgfsTer5	TC	4/41 (9.76%)	0/7	3/8	0/4	1/9	0/13
			Wild type	C	35/41 (85.37%)	6/7	5/8	4/4	7/9	13/13
*HYALP1*^23^	c.1298 T > C	Chr14:11,760,826	p.Met463Thr	C	1/56 (1.79%)	0/10	0/9	0/5	0/13	1/19
			Wild type	T	55/56 (98.21%)	10/10	9/9	5/5	13/13	18/19
*MYC*^24^	c.224C > T	Chr13:25,203,051	p.Ser75Phe	T	0/60 (0%)	0/11	0/12	0/5	0/13	0/19
			Wild type	C	60/60 (100%)	11/11	12/12	5/5	13/13	19/19
*LMNB1*^20^	c.920C > T	Chr11:16,038,811	p.Ser307Leu	T	0/60 (0%)	0/11	0/12	0/5	0/13	0/19
			Wild type	C	60/60 (100%)	11/11	12/12	5/5	13/13	19/19

Significant values are in bold.

*GR* Golden retriever, *LR* Labrador retriever, *ST* Shih Tzu.

To eliminate excess dNTPs from the previous step, 0.17 μL of 10X SAP buffer, 0.3 μL of SAP enzyme and 1.53 μL of HPLC-grade H_2_O were added to the step-one PCR products, to a total volume of 7 μL, and incubated in the thermal cycles of 37 °C for 40 min following by 85 °C for 5 min. The final step of the single-base extension reaction was performed with an IPLEX® Pro Reagent Kit (Agena Bioscience) to hybridize and elongate the extension primers at the nucleotide position of interest. For the single-base extension (ddNTPs), 0.2 μL IPLEX® Buffer (10X), 0.2 μL IPLEX® Terminator Mix (10X), 0.04 μL IPLEX® Pro Enzyme (33 U/μL) and 0.94 μL extension primers (0.58–1.21 μM) were mixed with the step-two products, and H_2_O was added to a total volume of 9 μL. The reaction was performed at 95 °C for 30 s, followed by five cycles of 95 °C for 5 s, 52 °C for 5 s and 80 °C for 5 s, for a total of 40 cycles, with a final extension at 72 °C for 3 min.

For desalination, 29 μL HPLC-grade H_2_O and 13 μL clean resin (96-well microplates) were added to the step-three extension products. Afterward, the supernatant was spotted onto a matrix-precoated SpectroCHIP® through a MassARRAY Nanodispenser and scanned using a MassARRAY Analyzer. The results were analyzed using MassARRAY Typer Software (v.4.1.8.3). The mutation was distinguished with TOF (time-of-flight) mass spectrometry on the basis of different molecular weights. The peaks in the mass spectrum were identified as mutations. Only those samples with a success rate greater than 80% were included in the analysis. Genotyping calls were viewed in call cluster plots, and peak intensities were reviewed in each respective sample spectrum. A SIFT (Sorting Intolerant From Tolerant) score from variant effector predictor in dog genome database was used to predict whether an amino acid substitution affects protein function (https://asia.ensembl.org/Multi/Tools/VEP).

### Data analysis

Kaplan–Meier survival analysis was used to analyze overall survival times (OST) from sex, breed, and chemotherapeutic protocol using GraphPad Prism version 9.2.0 (GraphPad Software, CA, USA). The SNP variants were selected to predict the OST and separately compared between the mutant and wild-type dogs received COP and CHOP. The *p*-value of survival analysis was calculated by medians of a log-rank test. The odds ratio of SNP location and dog breeds was calculated by Fisher’s exact test. *P* < 0.05 was understood to represent statistical significance.

## Results

### Study dogs

There were 63 dogs diagnosed as having nodal DLBCL, but three cases were excluded as they had call rates of less than 80%. Of the 60 remaining dogs, there were 47 purebreds and 13 crossbreed dogs (Table 1). The majority of the purebreds were golden retrievers (GRs), Shih Tzus and Labrador retrievers (LRs). The mean age of the dogs was 9.45 years old. Twenty-six dogs were males, sixteen were females, nine dogs were castrated males and nine dogs were spayed females.

### Gene polymorphisms’ detection

**Figure 1:**
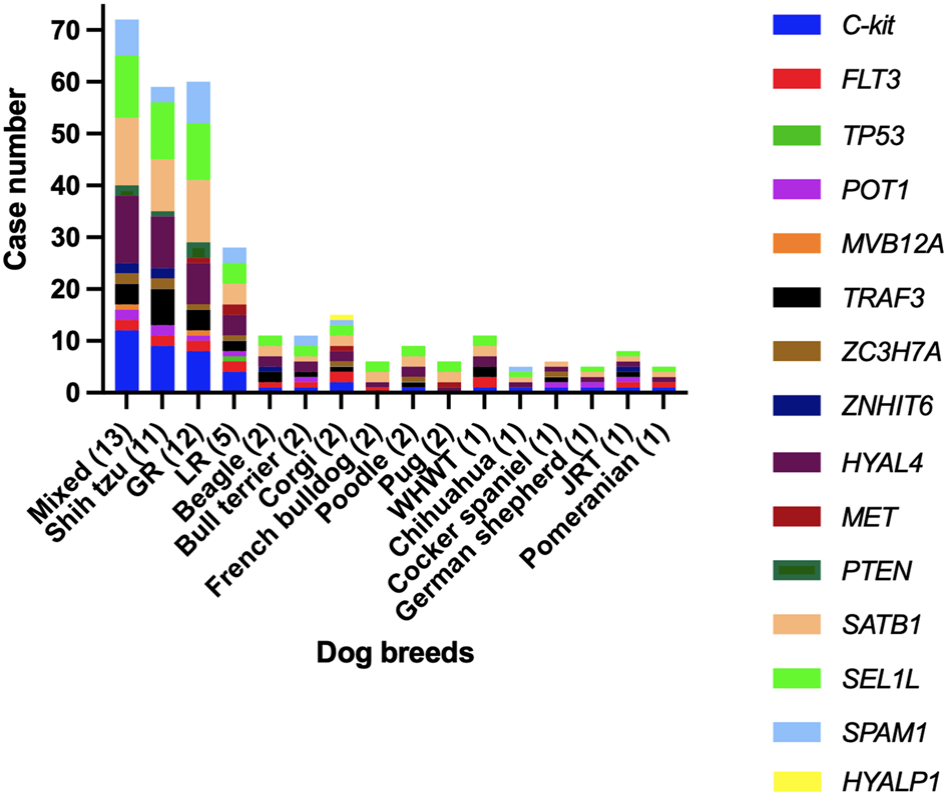
The bar chart shows the incidence of single nucleotide polymorphisms observed in each dog breed.

**Figure 2:**
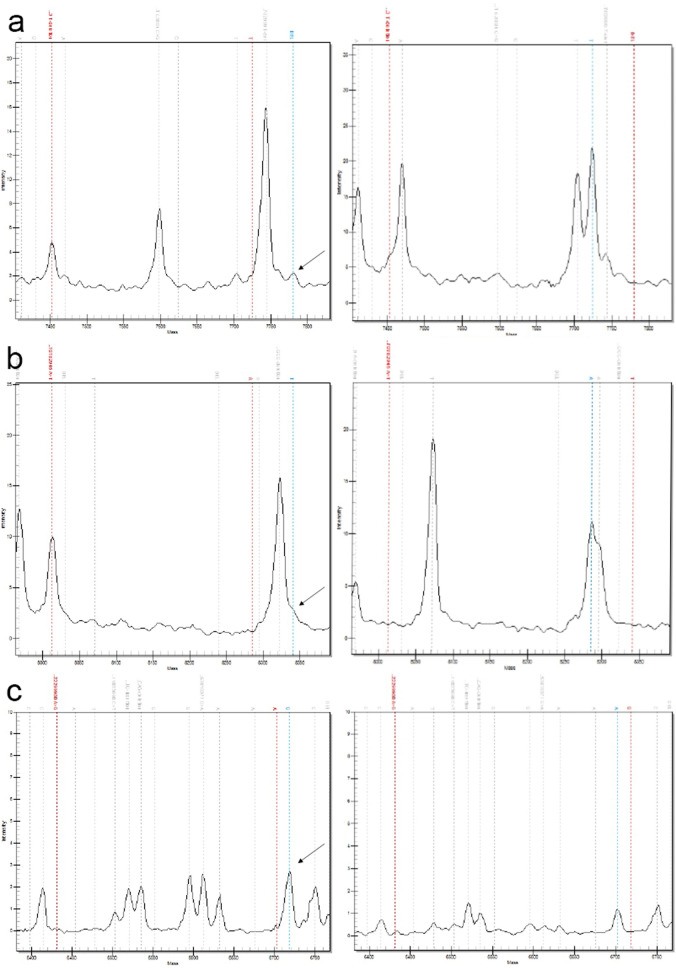
Example mutations depicted as mass spectra. (**a**) POT1 c.927del (p.Phe309LeufsTer3) exhibits T nucleotide deletion (arrow), (**b**) TRAF3 rs851689319 (p.Lys284Ter) presents nucleotide change from A to T (arrow), (**c**) c-KIT rs22299980 (p.Thr425 =) shows A to G substitution (arrow) compared to wild type .

Most of the primer variants had an efficiency of more than 80%, except for FLT3 rs23257447 T > C (63.3%), MET c.3804 C > G (66.7%), PTEN rs397513087 C > T (68.3%), SEL1L 8:53818371 G > A (43.3%) and ZC3H7A c.2792 A > G (16.67%). All dog breeds with DLBCL had mutations at SATB1^Q420P^ (c.1259 A > C, Gln420Pro) and HYAL4 (rs8499846, 14:11791385, 14:11794735 and 14:11807161) (Table 2 and Fig. 1). The lesser variants were observed at *c-Kit* T425 = (rs22299980 A > G, Thr425 =) and SEL1L (8:53778185, 8:53785948, rs24507594, 8:53818371 and rs24560262). The *c-Kit* wild type was only noted in two French bulldogs and two pugs, while wild-type *SEL1L* presented in one cocker spaniel. For the *POT1* and *TRAF3* mutations-i.e., the primary SNPs in canine DLBCL-four variants of POT1^R284C, F308X, R583*, F643S^ (c.850 C > T, c.927 del, c.1747 C > T and c. 1928 C > T) were accounted for 3–13% of all dogs and five variants of TRAF3^K284*, F306X, E303EX, ID323–324X, -316–317PKX^ (rs851689319, c.906del, c.908dup, c.968–971del and c.942-949dup) varied from 6–30% of all DLBCL cases (Table 2 and Fig. 2). In addition, when we compared the *FLT3* and *TRAF3* variants between dog breeds, purebreds tended to have these variants more than crossbreeds; the odds ratio for FLT3 rs852342480 was 3.88 (95%CI: 0.50–45.03, *p* = 0.26), for TRAF3^F306X^ was 3.64 (95%CI 0.58–42.50, *p* = 0.43) and for TRAF3^E303EX^ was 2.66 (95%CI 0.56–13.12, *p* = 0.31). No particular breeds were overrepresented in each SNP with any statistical significance.

### Survival analysis

**Figure 3:**
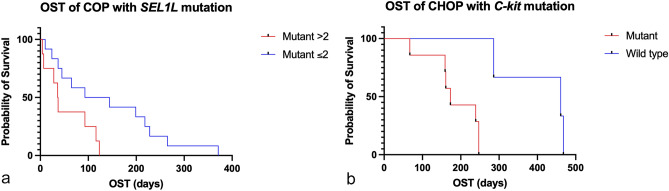
Kaplan–Meier survival analysis of overall survival time (OST) in DLBCL dogs treated with COP and CHOP protocols. (**a**) The median OST of COP-treated dogs with 3–5 variants on *SEL1L* was 37 days comparing to dogs with ≤ 2 variants on *SEL1L* (119 days, *p* = 0.03). (**b**) CHOP-treated dogs with mutant *c-Kit* had lower median OST than wild-type *c-Kit*. (173 and 461 days, respectively; *p* = 0.01).

Among 38 dogs receiving chemotherapy, eight dogs were loss of contact after treatment and only 30 patients were included in our survival analysis. One dog was deceased from heat stroke and others were deceased from lymphoma. The median OST of sex (male vs. female) and breed (purebred vs. crossbred) were not statistical difference. However, the median OST of 20 dogs treating with COP was lower than 10 dogs treating with CHOP (79 days vs. 243 days, *p* = 0.006). The median OST was calculated and compared between the mutant (*c-Kit*, *FLT3*, *POT1*, *TRAF3*, *HYAL4*, *SEL1L* and *SPAM1*) and wild-type dogs in two different protocols as shown in Table 3. For CHOP group, dogs with the *c-Kit* mutation had decreased median OST (173 days) when compared to those with the wild type (461 days), with a statistically significant difference (*p* = 0.01, Fig. 3b). For COP, dogs accumulated with three to five SNPs on *SEL11* had shorter median OST than dogs with ≤ 2 SNPs on *SEL11* (37 days vs. 119 days, *p* = 0.03, Fig. 3a).

**Table 3:** Survival analysis of selected mutant and wild type genes in 30 diffuse large B-cell lymphomas treated with COP- and CHOP-based chemotherapy.

Gene mutation	Median OST (days)	*p* value
**COP protocol (n = 20)**		
*C-kit* mutant (n = 17)	93	0.82
*C-kit* wild type (n = 3)	45	
**CHOP protocol (n = 10)**		
*C-kit* mutant (n = 7)	173	**0.01**
*C-kit* wild type (n = 3)	461	
**COP protocol (n = 20)**		
*FLT3* mutant (n = 2)	134	0.76
*FLT3* wild type (n = 18)	55	
**CHOP protocol (n = 10)**		
*FLT3* mutant (n = 3)	247	0.85
*FLT3* wild type (n = 7)	239	
**COP protocol (n = 20)**		
*TRAF3* mutant (n = 6)	105	0.76
*TRAF3* wild type (n = 14)	55	
**CHOP protocol (n = 10)**		
*TRAF3* mutant (n = 3)	239	0.28
*TRAF3* wild type (n = 7)	247	
**COP protocol (n = 20)**		
*POT1* mutant (n = 4)	146	0.36
*POT1* wild type (n = 16)	45	
**CHOP protocol (n = 10)**		
*POT1* mutant (n = 0)	–	–
*POT1* wild type (n = 10)		
**COP protocol (n = 20)**		
*HYAL4* mutant (n = 18)	93	< 0.0001
*HYAL4* wild type (n = 2)	5.5	
**CHOP protocol (n = 10)**		
*HYAL4* mutant (n = 2)	153	0.11
*HYAL4* wild type (n = 8)	247	
**COP protocol (n = 20)**		
*SEL1L* mutant > 2 locations (n = 8)	37	**0.03**
*SEL1L* mutant ≤ 2 locations (n = 12)	119	
**CHOP protocol (n = 10)**		
*SEL1L* mutant > 2 locations (n = 6)	210	0.59
*SEL1L* mutant ≤ 2 locations (n = 4)	243	
**COP protocol (n = 20)**		
*SPAM1* mutant (n = 8)	51	0.44
*SPAM1* wild type (n = 12)	93	
**CHOP protocol (n = 10)**		
*SPAM1* mutant (n = 5)	173	0.26
*SPAM1* wild type (n = 5)	247	

Significant values are in bold.

## Discussion

Our study developed a SNP panel of 41 locations and investigated in canine nodal DLBCL using a MassARRAY system. The MassARRAY panel could evaluate up to 40 variants from 192 samples in a single run and it is affordable in relation to other technologies available. The prevailing variants in DLBCL were SATB1 Q420P (96.67%), c-Kit rs22299980 (75.86%) and SEL1L 8:53778185 (74.58%). Among three locations, SATB1^Q420P^ had a moderate impact on the protein function, with a SIFT score equal to 0.0 (deleterious). In contrast, a nonsense mutation of *c-Kit* had a low impact and an intron variant of *SEL1L* was a modifier.

*Special AT-rich sequence-binding protein 1* (*SATB1*) is a global transcription regulator and chromatin organizer. *SATB1* encodes a binding nuclear matrix protein that recruits the chromatin remodeling factor, to regulate the chromatin structure and gene expression. It regulates the gene expression in thymocytes and pre-B cells^29^. As mentioned in a previous report, this gene was mutated in 12–25% of T-cell lymphoma cases in boxers and golden retrievers^18^. Harris et al*.*^22^ also found that one non-boxer dog with PTCL had SNP at c. 1259 T > G in *SATB1*^L420^^R^. The author selected this SNP location followed aforementioned studies; however, different mutation (c. 1259 A > C) was used in this SNP panel. The polymorphisms of *SATB1*^Q420P, Q420PX^ were observed in all purebred and mixed-breed dogs with DLBCL. In human cancers, this gene promotes tumor progression and metastasis in breast cancer, colorectal cancer and cutaneous T-cell lymphoma^30–32^. As the authors could not indicate the significance of mutated *SATB1* and its expression in high-grade nodal lymphomas in dogs, further study is required to confirm the significant pathogenesis, and a target drug that could restore *SATB1* function might be helpful for the treatment of canine lymphomas.

The c*-Kit* proto-oncogene is encoded as the transmembrane type-III receptor tyrosine kinase KIT, which is expressed in the hematopoietic progenitor of both myeloid and lymphoid cells. It plays a role in proliferation, cell survival and differentiation of hematopoietic precursors^33,34^. As *c-Kit* mutations are mainly reported in canine mast-cell tumors, few studies have investigated its mutation in canine leukemia and lymphoma. A nonsense mutation at codon 425 of exon 8 (rs22299980 A > G, Thr425 =) was observed in 73% (30/41) of dogs with acute myeloid leukemia^35^, and a similar polymorphism of *c-Kit* T425 = was described in mixed-breed dogs with lymphoma^36^. Although the *c-Kit* mutation was rarely reported in canine nodal lymphomas, we decided to choose the SNP location of the *c-Kit* gene and investigate in canine DLBCL with an increased sample size. Surprisingly, 44 dogs (75.86%) with DLBCL, from most breeds except French bulldogs and pugs, had mutated *c-Kit*. Synonymous variants of *c-Kit* T425 = were enriched in DLBCL, followed by frameshift variant of *c-Kit* T425TX (rs22299980 A > AG). However, the significance of the frameshift mutation in canine lymphomagenesis was undetermined in this study. Yamazaki et al*.*^37^ investigated the response rate of toceranib phosphate, one of the tyrosine kinase inhibitor (TKI) drugs used to treat unresectable high-grade mast-cell tumor with *c-Kit* mutation, in dogs with refractory T-cell lymphoma. The overall response rate of this monotherapy was only 40% in three dogs with partial remission and two dogs with stable disease. Therefore, the effectiveness and efficacy of TKI to treat canine lymphoma with *c-Kit* T425 = /T425TX require further investigation. In addition, *c-Kit* mutation has a significant prognosis in canine cutaneous mast cell tumors. Internal tandem duplication in exon 11 were approximately observed in 30–50% of higher grade mast cell tumors^38^. It has been associated with decreased survival times and progression-free survivals^39,40^. No evidence has reported on *c-Kit* mutation and its prognostic value in canine lymphoma. As such, its significance on prognosis needs further investigation in canine DLBCL.

The common SNP locations identified in canine DLBCL were *POT1* and *TRAF3*^18^. The telomere-binding protein protection of telomeres 1 (POT1), encoded by *POT1*, serves as providing telomere maintenance, and its dysfunction can lead to defective telomere replication, in turn, leading to genomic instability and enhanced carcinogenesis^41^. The adaptor protein TNF receptor associated factor 3 (TRAF3) is a tumor-suppressor gene that plays a critical role in B lymphocyte survival. A *TRAF3* mutation leads to upregulation of the NF-kB pathway^19^. Both *POT1* and *TRAF3* mutations have been reported in human and canine B-cell lymphoma^18,19,21^. Elvers et al*.*^18^ found *POT1* and *TRAF3* mutations in 17–20% of B-cell lymphoma cases in both golden retrievers and cocker spaniels. Therefore, our study selected SNP mutations of the POT1 and TRAF3 genes, following Smith et al*.*^21^. Each dog with a *POT1* mutation tended to have more than one enriched variant, with a similar observation in *TRAF3*. Therefore, the novel target drugs against POT1 and TRAF3 might have therapeutic potential for treating DLBCL in dogs.

As lymphoma-risked genes depend on hereditary or somatic mutations, the best way to determine the significant genes is in one population, such as in a predisposing breed like boxers or retriever dogs. Our study focused on the application of the MassARRAY technique to detect nominated lymphoma variants from a valuable database in various dog breeds with the DLBCL subtype. Thus, it was possible that the candidate SNPs were not discovered in some dogs or were discovered at lower rates than usual. Moreover, few primers of each SNP location in our study had an amplification efficiency lower than 60%, especially in *ZC3H7A*^H931R^. Although the true prevalence could not be determined in our study, nine out of ten dogs (90%) had a mutation in *ZC3H7A*^H931R^. The missense mutation of *ZC3H7A* was also reported in canine DLBCL, with predictable functional consequences^20^.

In summary, the SNPs panel of one variant/gene (*C-kit*, *SATB1*, *TP53*, *MVB12A*, *ZC3H7A*, *ZNHIT6*, *MET*, *PTEN*, *HYALP1*, *MYC*, and *LMNB1*), three variants/gene (*FLT3* and *SPAM1*), four variants/gene (*POT1* and *HYAL4*), five variants/gene (*SEL1L*), and 11 variants/gene (*TRAF3*) were determined in dogs with diffuse large B-cell lymphoma. Most of the variants could be detected in both purebred and mixed-breed dogs, excluding MYC^S75F^, TRAF3^A531VX, L399X, D551X, T477X, VIDSQA322–327VX, MRGEY478–482I^ and LMNB1^S307L^. The application of the MassARRAY technique to discover SNPs provides informative data on each subject and could be used to identify a prognosis and develop a treatment strategy for each lymphoma dog.
